# Validation of novel patient-centred juvenile idiopathic arthritis-specific patient-reported outcome and experience measures (PROMs/PREMs)

**DOI:** 10.1186/s12969-020-00481-2

**Published:** 2020-11-19

**Authors:** Laura E. Lunt, Stephanie Shoop-Worrall, Nicola Smith, Gavin Cleary, Janet McDonagh, Andrew D. Smith, Wendy Thomson, Flora McErlane

**Affiliations:** 1 Versus Arthritis Centre for Epidemiology, Centre for Musculoskeletal Research, The University of Manchester, Manchester, UK; 2 National Institute of Health Research Manchester Biomedical Research Centre, Manchester Academic Health Science Centre, Manchester University NHS Foundation Trust, Manchester, UK; 3 Centre for Health Informatics, The University of Manchester, Manchester, UK; 4 Musculoskeletal Research Group, Institute of Cellular Medicine, Newcastle University, Newcastle Upon Tyne, UK; 5 Department of Rheumatology, Alder Hey Children’s Hospital, Liverpool, UK; 6 Royal Manchester Children’s Hospital, Manchester University Hospitals Trust, Manchester, UK; 7 Centre for Genetics and Genomics Versus Arthritis, Centre for Musculoskeletal Research, The University of Manchester, Manchester, UK; 8 Department of Paediatric Rheumatology, Great North Children’s Hospital, Queen Victoria Road, Newcastle upon Tyne, NE1 4LP UK; 9 Institute of Population and Health Sciences, Medical School, Newcastle University, Newcastle upon Tyne, UK

**Keywords:** Juvenile idiopathic arthritis, Quality of care, Patient-reported outcomes, Validation, OMERACT

## Abstract

**Background:**

Measuring the outcomes that matter to children and young people (CYP) with juvenile idiopathic arthritis (JIA), is a necessary precursor to patient-centred improvements in quality of clinical care. We present a two-centre validation of novel JIA patient-reported outcome and experience measures (PROM and PREM) developed as part of the CAPTURE-JIA project.

**Methods:**

CYP with JIA were recruited from paediatric rheumatology clinics, completing the CAPTURE-JIA PROM and PREM, CHAQ and CHU 9D. A subset participated in face-to-face interviews and completed the PROM/PREM 1 week later. The OMERACT filter was applied and the three domains of validation assessed. Truth assessments included cognitive interviewing, sensitivity analysis and Spearman’s correlations. Discrimination assessments included specificity and reliability testing. Feasibility was assessed using time to form completion and proportion of missing data.

**Results:**

Eighty-two CYP and their families were recruited; ten cognitive interviews and fifteen PROM/PREM test/retests were conducted. Truth: CYP and parents understood the PROM/PREM and felt important areas were covered. PROM criteria had high sensitivities (> 70%) against similar items on the CHU 9D, with the exception of fatigue (58%). Correlations between similar PROM and CHU 9D criteria were moderate to very strong (coefficients 0.40–0.82.) Discrimination: high specificities (> 70%) on corresponding PROM and CHU 9D domains. Feasibility: median completion times for PROM 60 s (IQR 38–75) and PREM 49 s (IQR 30–60) respectively.

**Conclusion:**

The CAPTURE-JIA PROM and PREM are valid and feasible in UK paediatric rheumatology clinics. Embedding routine collection into clinical care would be a major step towards improving quality of care.

**Supplementary Information:**

The online version contains supplementary material available at 10.1186/s12969-020-00481-2.

## Key messages

- Measurement of patient-reported outcomes is central to improving quality of care in juvenile idiopathic arthritis.
- The CAPTURE-JIA PROM and PREM are valid, feasible and acceptable in UK paediatric rheumatology clinics.

## Background

Juvenile idiopathic arthritis (JIA) is an umbrella term for a heterogeneous group of conditions characterised by inflammatory arthritis and onset before the age of 16 years [1, 2]. JIA is one of the most common chronic inflammatory diseases of childhood [3, 4] and can have a major impact on the life of the child/young person/CYP, affecting physical (including visual function), psychological, health-related quality of life (HRQoL) and social/educational attainments [5–7]. Persistent joint inflammation can lead to fatigue, pain, growth disturbances, joint damage and joint deformity [5, 8]. Chronic ill health and functional limitation are associated with poorer health related quality of life (HRQoL) and behavioural problems [9]. These effects are known to persist into adult life, profoundly affecting generic health status, quality of life and employment rates [7, 10]. JIA can also impact on the productivity and quality of life of the wider family, with parents reporting significant work-time loss and out-of-pocket costs [11].

Over the past 10 years, a concerted international effort has led to the development of a wide range of composite disease activity and outcome instruments specific to JIA [12]. These instruments are increasingly used as primary and secondary outcome measures in JIA clinical trials and have had a significant impact on our understanding of clinical outcomes; for example, recent observational studies consistently suggest that complete disease control (or inactive disease) is not routinely achieved in the clinical setting [13]; in fact, routine clinical outcomes in JIA lag considerably behind those from clinical trials employing intensive or targeted early treatment regimes [6, 14–16].

Many of these modern disease activity instruments include patient/parent-reported variables; for example, the JIA Core Outcome Variables include a self-reported functional assessment (the Childhood Health Assessment Questionnaire (CHAQ) and a 0-10 cm visual analogue scale (VAS) for self-reporting of global well-being [17]. These assessment tools were designed by research teams to assess areas of concern highlighted by clinicians. Anecdotally, CYP and families struggle to use these tools to directly report the constructs important to them in their everyday lives.

Measuring the outcomes that matter to CYP and their families (for example persistent symptoms, medication side-effects, function and quality of life), either in the context of clinical research or quality improvement exercises, is a necessary precursor to patient-centred improvements in the quality of clinical care and service delivery. Patient-reported outcomes present particular challenges in the paediatric setting and, where possible, must be designed to collect reports from both the child/young person and the parent [18]. One way to measure the outcomes that matter is to use well-designed and validated patient-reported outcome measures (PROM) and patient-reported experience measures (PREM). PROM and PREM contain health-related information reported directly by the patient, without interpretation by another individual (for example a researcher or member of the clinical team) [19]. To ensure that the most relevant outcomes are captured, involvement of the target population from conception onwards is considered essential [19]. Although PROMs are well-established quality improvement tools, understanding the patient experience and how it relates to outcomes is a relatively new concept. PREMs provide feedback on the quality of the overall care delivered to an individual patient, important from both quality improvement and research perspectives.

A 2013 multi-centre UK audit against the British Society for Paediatric and Adolescent Rheumatology (BSPAR)/Arthritis and Musculoskeletal Alliance (ARMA) Standards of Care (SOC) for children and young people (CYP) with JIA [20], highlighted the need for consensus agreed and measurable patient-reported JIA-specific outcome measures, to enable standardisation of clinical data collection and an improved understanding of the impact of variations in care on outcomes in JIA [21]. In response, our group developed a clinically relevant and feasible core dataset for JIA (termed ‘CAPTURE JIA’) including complete patient information relevant to disease outcomes, service delivery and clinical research [22]. The most relevant and clinically feasible patient/parent-reported outcome and experience measures were prioritised and selected using a modified nominal group approach with national consumer-led community involvement. No pre-existing questionnaire was able to capture the complete range of themes identified as important by the CYP or parent/carers. The clinician and patient/carer reported data items required to evaluate the national clinical audit questions were therefore collated and novel CAPTURE-JIA PROM and PREM questionnaires developed [23].

Robust and evidence-based validation of the CAPTURE-JIA PROM/PREM tools is key to the completion of the national audit project and an essential precursor to national PROM/PREM collection and analysis. Validation is the process of evaluating a new tool to guarantee it is measuring the intended variables, is acceptable and feasible in the clinical environment. The OMERACT filter is a recognized guide to outcome measurement validation [24].

### Aims and objectives

The aim of this mixed-methods study was to validate and pilot the CAPTURE-JIA PROM and PREM using the OMERACT filter in a two-centre patient population.

## Methods

Details of the consensus-based development of the CAPTURE-JIA PROM and PREM questionnaires have been described elsewhere [23]. In brief, the PROM comprises three core themes; physical, social and emotional wellbeing. All questions relate to a four-point response scale ranging from never (score = 0) to most of the time (score = 3). Questions relate to the past month. The PREM refers to the patient’s experience of the clinical encounter and encompasses the following themes; communication, information/education, environment and access/coordination of care. Response scales devised for questions 1 to 4 range from not at all (score = 0) to fully (score = 3). The response scale for question 5, addressing appointment delay, ranges from no unacceptable delay (score = 0) to unacceptable > 2 h delay (score = 5). There are two versions of both PROM and PREM; questionnaires for CYP aged < 11 years are completed by the parent/carer whilst questionnaires for CYP aged 11 years or over are completed by the patient.

Validation included both quantitative and qualitative approaches in accordance with the OMERACT filters. Part One of the study consisted of cognitive interviews eliciting opinion from study participants and Part Two included participant completion of the CAPTURE-JIA PROM and PREM questionnaires. The Child Health Utility 9D (CHU 9D) [25], a validated measure capturing similar themes to those included on the PROM, was used as a reference measure for the PROM. The PREM is a unique tool, there are no JIA-relevant patient experience tools for comparison.

The study complies with the Declaration of Helsinki, the locally appointed ethics committee approved the research protocol [National Research Ethics Committee East Midlands-Leicester IRAS 212656] and informed consent was obtained from all subjects (or their legally authorized representative).

### Part one

#### Study population

A convenience sample of CYP with a confirmed diagnosis of JIA attending paediatric rheumatology clinic between September and November 2017 were invited to participate in the study. Children < 1 year of age and families not fluent in English were excluded.

#### Data collection

Three rounds of cognitive interviews were conducted in a private room in the paediatric rheumatology clinic by an experienced female qualitative research assistant (post PhD). Interviews lasted an average of 20min and sampling continued until data saturation was achieved. Think aloud techniques were used to elicit opinions on the PROM and PREM questionnaires, with parents/patients (as relevant depending on their age) invited to read each question aloud, explain their understanding of the question and describe any areas which they felt lacked clarity. Areas identified as unclear were probed in detail and parents/patients asked to suggest improvements. At the end of the interview, families were encouraged to identify any additional and relevant topics or issues for discussion. Interviews were audio-recorded with the participants consent, transcribed (and edited to ensure anonymity of respondent), and transcripts formed the data subjected to formal analysis. Data were analysed qualitatively by one experienced researcher and conducted according to the standard procedures of rigorous qualitative analysis [26], using procedures from first-generation grounded theory (coding, constant comparison, memoing) [27], from analytic induction (deviant case analysis) [28] and constructionist grounded theory (mapping) [29]. Data collection and analysis occurred concurrently, so that issues raised in earlier rounds of fieldwork could be explored subsequently.

Reflexivity was maintained by the research team throughout analysis and writing, by recording, discussing and challenging established assumptions. Joint first author NS conducted and analysed all interviews. Although she has a wide range of experience with JIA families, she was not known to the participants of this research prior to undertaking the study and was based in an external setting. This ensured she held no preconceptions in relation to health service delivery and gave participants the opportunity to discuss their thoughts without any potential influence from their care team.

### Part two

#### Study population

CYP with a confirmed diagnosis of JIA attending paediatric rheumatology clinic at the Great North Children’s Hospital in Newcastle or Royal Manchester Children’s Hospital in Manchester between September 2017 and February 2018 were eligible for inclusion. Children < 1 year of age and families not fluent in English were excluded.

#### Data collection

Participants were asked to complete the PROM and CHU 9D in the hospital waiting area before the clinical consultation and to complete the PREM after the consultation had taken place. A subset of recruited participants were asked to complete the PROM and PREM 1 week later at home, returning the completed forms to the research team in a stamped addressed envelope. Participants were recruited over a period of 6 months. All data were stored at the University of Manchester in accordance with data governance regulations.

### Statistical analyses

The OMERACT filter was applied to assess three core domains of measurement validation; truth, discrimination and feasibility [30, 31]. The PROM was validated against the CHU 9D at each stage of validation with the exception of ‘*medication side effects’* which is not captured on the CHU 9D. For the majority of the validation techniques, raw scores of each measure were used. For sensitivity and specificity assessments, outcome scores were dichotomised to high (‘often’ and ‘most of the time’ on the PROM; ‘quite’ and ‘very’ or, ‘many’ and ‘I can’t’ on the CHU 9D) and low scores (‘never’ and ‘sometimes’ on the PROM; ‘I don’t’, ‘a little bit’ and ‘a bit’ or, ‘no problems’, ‘a few problems’ and ‘some problems’ on the CHU 9D). Since the PREM is a unique tool, assessment against the OMERACT filter was limited.

### Truth domain

This first domain of the OMERACT filter assesses whether each criterion is measuring what it is intended to measure, in an unbiased way. It encompasses face, content, criterion, and construct validity.

#### Face validity (PROM and PREM)

Aims to provide evidence the criteria included on the measure is sensible, relevant and comprehensive. Can the CYP/parents completing the measure understand the criteria and what is being asked? Are the themes important and do they address areas of important relevance to the CYP/parent?

#### Content validity (PROM and PREM)

do CYP/parent understand the questions correctly and provide answers using suitable rating scales? Qualitative analysis of the cognitive interview transcripts provided insight into what CYP/parents understood by the questions. Within the cognitive interviews, CYP/parents were further given the opportunity to comment on the relevance of the themes within the questions and identify items important to them personally.

#### Criterion validity (PROM)

Investigates whether patients are classified in the same way by the new measure as a previously validated measurement tool capturing the same or similar constructs. This was tested by assessing the sensitivity of the PROM in identifying high symptom levels in each domain versus high symptom levels in corresponding CHU 9D domains.

#### Construct validity (PROM)

To assess how well each PROM criterion measures the intended underlying constructs, we evaluated convergence with similar criteria on the CHU 9D (Spearman correlations.)

### Discrimination domain (PROM and PREM)

#### Classification validity

Can each criterion on the PROM identify whether or not a patient has the symptom of interest? Specificity of low scores on the PROM were tested against low scores on the CHU 9D. Area under the curve (AUC) analyses using receiver operating characteristics enabled determination of levels of distinction for classifying high from low symptom levels.

#### Reproducibility of results

Test re-test reliability used linear-weighted kappa coefficients to assess the strength of agreement of the ordinal scores completed 1 week apart.

### Feasibility domain (PROM and PREM)

This element of the OMERACT filter assesses how easily the measures can be applied in the intended environment. (A hospital waiting area before and after the clinical consultation.) Time taken to complete the PROM, PREM and CHU 9D was recorded using a stopwatch and the proportion of participants completing each item on the PROM, PREM and CHU 9D was calculated. A cut-off of 80% completion was selected for data items to be considered feasible in the clinical environment.

## Results

### Study characteristics

Eighty-two CYP with JIA/parent were recruited; ten completed face-to-face interviews and seventy-two completed the PROM, PREM and CHU 9D. Interviews lasted on average approximately 11.5 min (Mean: 11 min, 34 s, Range: 6 min, 53 s – 22 min, 27 s). Fifteen of twenty participants approached returned the PROM and PREM forms 1 week later (75%) (Table 1). A complete case analyses approach was taken.

**Table 1:** Study characteristics

Questionnaire	Number of data items	N (%) Completed questionnaires	N (%) Form type		Median time to completion in seconds (IQR)^a^
			< 11 yrs	11 yrs. >	
PROM	6	72 (100%)	51 (71%)	21 (29%)	60 (38–75)
PREM	5	72 (100%)			49 (30–60)
CHU 9D	9	69 (95%)	–	–	74 (60–91)

^a^ Based on number of observations with completion time recorded

### Truth domain

#### Face validity

Qualitative analysis of the cognitive interview transcripts confirmed families understood what each item of the PROM and PREM questionnaires was asking. The PROM questionnaire provided families with the opportunity to respond in a manner they felt appropriate and using a suitable scale system so no changes were made to the wording of the original questionnaire. The majority of families identified that Question 5 asked for a single response to two related data items – length of delay and whether the delay was acceptable. Families reported that delay is not always unacceptable and therefore considered it important to distinguish between the two. In response, Question 5 was separated into two related questions (Question 5 and Question 6).

#### Content validity

Comments within the transcripts further confirmed that the main issues important to each family were covered within the PROM and PREM items. It was clear that patient/parent pairs felt all the items included were not only factors of concern to them personally but also relevant to the JIA population as a whole. With this in mind, both tools appeared comprehensive measures based on each family’s personal experiences.

Further analysis of the interview transcripts revealed four points for consideration. Upon reflection, no further amendments to the existing questionnaire were considered necessary, although development of a short patient /parent completion guide may be helpful. The questionnaire is purposefully brief and families unanimously agreed that it should not be lengthened unnecessarily (Table 2).

**Table 2:** PROM / PREM data items highlighted for further discussion during the face-to-face interviews

Suggested Data Item	Discussion Points	Amendment Yes or No
School	Some families queried the addition of a specific question on school. After careful consideration, it was decided that school-related concerns should be addressed within the existing data items.	No
Mobility	One parent queried the need to include a specific PROM item on mobility. Other families considered this suggestion but did not agree. Mobility is assessed in detail in the CHAQ assessment.	No
Dressing/ Undressing	One of the families highlighted the importance of dressing/undressing capability and queried whether an additional question may be required. After careful consideration it was decided that the physical well-being item within the questionnaire is sufficient to capture dressing/undressing. Dressing/undressing is explored in the CHAQ assessment.	No
Free Text Boxes	The option to expand on answers was suggested as a useful revision for both questionnaires. PROM: the opportunity to note why the child was reporting a particular outcome was suggested. PREM: the opportunity to identify why the consultation was rated poorly was suggested as an important precursor to implementation of improvements. Further discussion within the research team clarified that the addition of free text format would not be in line with the initial aims of the PROM PREM development; rather clinicians should be encouraged to explore the reasoning behind responses within or following consultations as relevant.	No

^a^ Further analysis of the interview transcripts revealed four points for consideration. Upon reflection, no further amendments to the existing questionnaire were considered necessary

### Criterion validity

Overall, sensitivities were high for the majority of PROM items in comparison with similar items on the CHU 9D. (4 items > 70%.) The only exception was ‘*daily activities interfered by fatigue’* on the PROM, which had sensitivity of 58% against ‘*feeling tired’* on the CHU 9D (Table 3).

**Table 3:** Sensitivity and specificity of PROM and CHU 9D items

Questionnaire item	Sensitivity	Specificity
PROM fatigue	58.30%	90.70%
CHU9D tired		
PROM pain	75%	71.40%
CHU9D pain		
PROM sleep	100%	78.70%
CHU9D sleep		
PROM social	100%	87.60%
CHU9D activities		
PROM emotional	100%	78.70%
CHU9D worry		
PROM emotional	100%	76.40%
CHU9D sad		
PROM emotional	100%	77.60%
CHU9D annoyed		

### Construct validity

Significant positive correlations were evident between all items on the PROM and CHU 9D (r range 0.37 to 0.83, *p* < 0.05) (Table 4).

**Table 4:** Spearman correlation coefficients

		CHU 9D data items						
		Pain	Social activities	Problems sleeping	Sad	Worried	Annoyed	Tired
PROM data items	Pain	0.83						
	Social wellbeing		0.64					
	Poor sleep			0.62				
	Emotional wellbeing				0.58	0.52	0.37	
	Fatigue							0.44

### Discrimination domain

**Fig. 1:**
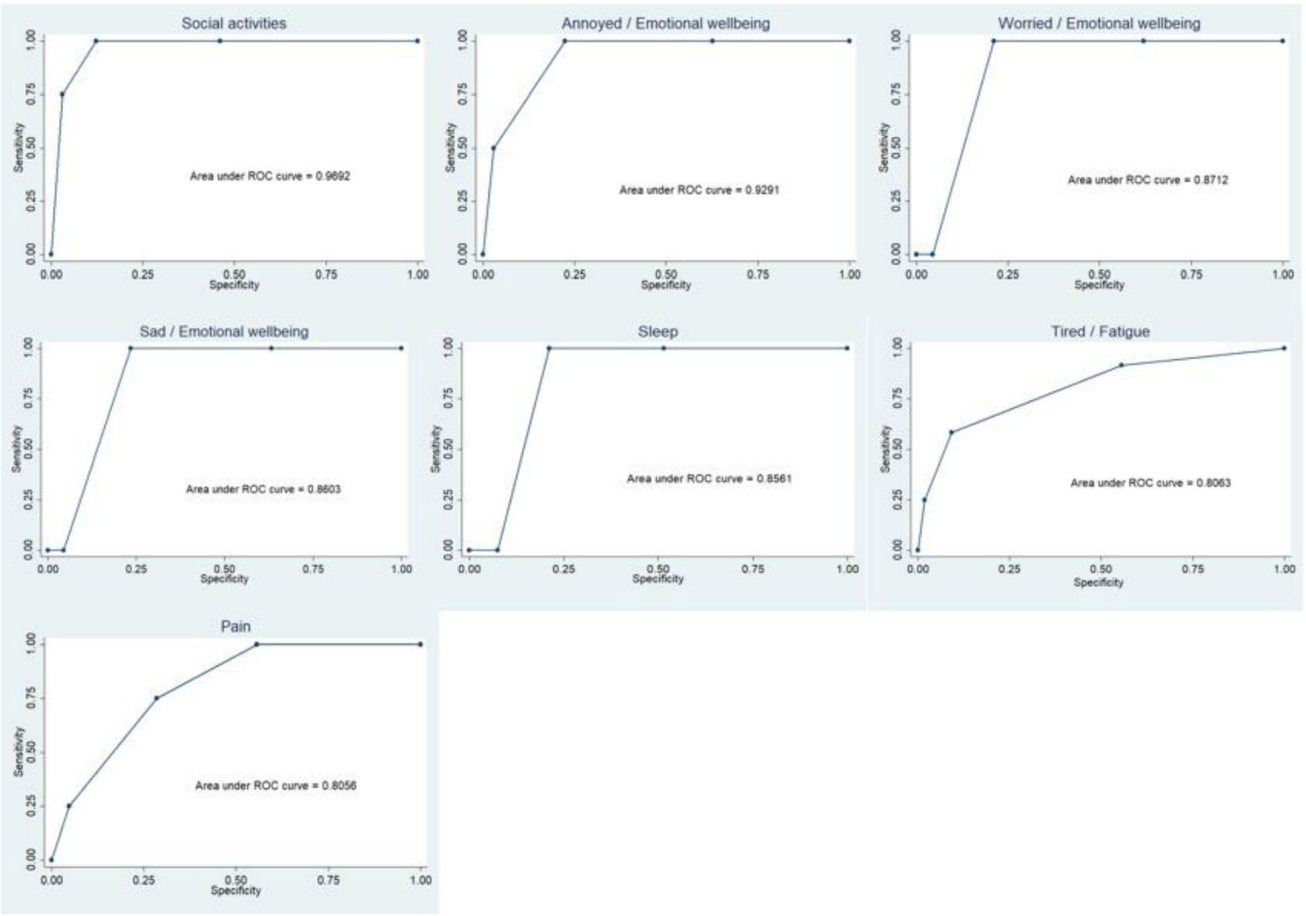
ROC Curve analysis. * Area under the curve values using ROC curve analyses ranged from 0.80 to 0.96, indicating that all items on the PROM could distinguish well between patients with and without the symptom

There was high specificity between criteria on the PROM (5 items > 70% specificity) and relevant items on the CHU 9D (Table 3). Further, AUC values using ROC curve analyses ranged from 0.80 to 0.96, indicating that all items on the PROM could distinguish well between patients with and without the symptom (Fig. 1). Moderate to substantial test re-test reliability of all PROM items was demonstrated (kappa coefficients range 0.50 to 0.71) (Table 5). The majority of scores on the PREM when completed in clinic were identical to scores when completed 1 week later at home. Thus calculating Kappa coefficients in this sample was not appropriate.

**Table 5:** Linear-weighted Kappa coefficients

PROM items	Agreement %	Strength of agreement (kappa coefficient)	kappa coefficient range
Pain	91	0.71	Substantial (0.61–0.80)
Social wellbeing	91	0.71	
Medication side effects	86	0.63	
Emotional wellbeing	87	0.62	
Fatigue	86	0.57	Moderate (0.41–0.60)
Poor sleep	86	0.50	

^a^ Kappa coefficients ranging from 0.50 to 0.71 indicate moderate to substantial test-retest reliability

### Feasibility domain

Median completion times for the PROM and PREM were 60 s (IQR 38–75) and 49 s (IQR 30–60) respectively. In comparison, the CHU 9D took a median of 74 s to be completed (IQR 60–91) (Table 1). All items on the PROM and PREM obtained at least 90% completion.

The updated PROM and PREM questionnaires are included as Supplementary Tables S1 and S2.

## Discussion

The CAPTURE-JIA PROM and PREM tools are valid and feasible in UK paediatric rheumatology clinics. They are the first outcome tools to be developed by the UK patient and parent community. Items included on each measure are clinically relevant, key to service delivery auditing and deemed important and relevant by the patient and parent community. Both measures could be applied to other health disciplines making the utility and adaptability of these tools universal.

The PROM questionnaire was designed to be a new and unique tool measuring the outcomes that matter to families. Our consumer group told us that these important outcomes include persistent symptoms, medication side-effects, function and quality of life. The questions were devised and worded in conjunction with parents and young people to ensure that they asked the questions that families wanted to be asked. Since this is a novel approach to development of a JIA specific PROM, there are no pre-existing similar tools. Although the CHU 9D and PROM appear to capture similar information, there are important differences between the two measurement tools. The CHU 9D was designed as a generic research tool whereas the PROM was designed by JIA patients and families to address specific areas of concern in the context of busy clinical assessments. The PROM assesses quality of life over a longer time period (1 month versus 1 day); families of CYP with JIA reported significant day-to-day variation in symptoms and specifically requested a measurement tool assessing quality of life over the past month. The PROM was purposefully designed to be a simple tool fit for use in the busy clinical setting and, as a consequence, took less time to complete than the CHU 9D (60 versus 74 s). Neither tool required modification of any data item; involving CYP and their families throughout the design process was likely a key factor in the success of our early methodological approach.

Application of the OMERACT filter demonstrated that the PROM captures the themes it is designed to measure, ensuring that information important to CYP and their families can be correctly identified and reported. The lower sensitivity between *daily activities interfered by fatigue* on the PROM and *feeling tired* on the CHU 9D was of interest; it is likely that the lower sensitivity reflects a difference between the concept and interpretations of fatigue versus tiredness. CYP and families designing the questionnaire specifically requested inclusion of the word ‘fatigue’. They felt strongly that ‘tiredness’ is another term for ‘sleepy’. Fatigue (defined as ‘extreme tiredness resulting from mental or physical exertion or illness’) was identified as the term that most closely described how CYP felt.

Further analyses indicated the PROM could accurately identity participants with low scores on all criteria. Positive correlations between individual items on the PROM and CHU 9D provide important evidence that the PROM fulfils the truth domain of the OMERACT filter. The term *emotional wellbeing* correlates better with sadness and worry than annoyance. This is interesting information, suggesting that families may not always consider feelings of annoyance relevant to overall emotional wellbeing. A future study could explore the constructs contained within emotional wellbeing in more detail; this information could be relevant to psychologists and other professionals working to improve resilience.

High test-retest reliability of the PROM indicates that each individual item is presented to the patient in a clear way and in turn understood. Although reliability testing of the PREM in this study was not appropriate (due to identical scoring), this may be less relevant as PREM data is more reflective in nature. For example, if a patient experiences a delay considered unacceptable at the time, the delay may become less significant as time elapses.

The approach taken to study design is one of the key strengths of this research. The OMERACT filter is a recognisable and widely accepted outcome validation framework. Application of both qualitative and quantitative research methods enabled a rich depth of information relating to clinical feasibility, accuracy and reliability.

Both the PROM and the PREM are relatively short and straightforward to complete, with at least 90% of participants completing each item on the individual tools. In comparison with these new tools, the CHAQ can take up to 10 min to complete [32] although shorter completion times are commonly reported [33]. CYP report that the CHAQ is too long and does not capture the lived experience of rheumatic conditions [34]. Ease of completion is a vital in a clinical waiting environment, which may be busy, noisy and full of distractions, and was a key ambition for CYP and families involved at each stage of questionnaire development.

The unique nature of the PREM is both a strength and a limitation of this validation effort. To our knowledge, the PREM is the first measurement tool designed by CYP and parents to focus on the JIA patient’s experiences in the clinical environment, providing families with a novel opportunity to share key experiences and enable assessment of the quality of local services. Although this is a strength of the study, the absence of relevant existing measures prevented validation of the PREM across all OMERACT filters, resulting in an inconclusive assessment of concurrent validity.

At present, there is wide variability in the completeness of patient-reported data collection in JIA [35]. Previous work has clearly demonstrated important differences between physician-reported disease activity data (such as joint counts/physician global scores) and patient-reported outcomes (such as global wellbeing scores), with a quarter of children and young people in clinical remission experiencing ongoing symptoms [13]. Routine clinical collection of key patient reported data items would add important information to existing knowledge about the impact of JIA on the everyday lives of CYP and their families.

This validation study demonstrated that the PROM and PREM questions are clearly written in a way that can be readily understood by UK patients and parents. Further work exploring how the questionnaires function in different demographic groups, ILAR subtypes and levels of disease activity will be an essential aspect of routine clinical adoption.

## Conclusion

The CAPTURE-JIA PROM and PREM are valid, feasible and acceptable to CYP / families with JIA attending UK paediatric rheumatology clinics. Items included on each measure are clinically relevant, key to service delivery auditing and deemed important and relevant by the patient and parent community. Routine clinical collection of data items prioritised as important by patients and their families, in addition to key disease activity data items, would be a major step towards understanding and subsequently improving the quality and consistency of clinical services across the UK.

## Supplementary Information

**Additional file 1:** **Supplementary Table S1.** Patient Reported Outcome and Experience Measures for Children Aged < 11 Years. **Supplementary Table S2.** Patient Reported Outcome and Experience Measures for Young People Aged ≥ 11 years.

## Data Availability

Not applicable for this study.

## Abbreviations

JIA: Juvenile idiopathic arthritis
PROM: Patient reported outcome measure
PREM: Patient reported experience measure
CYP: Child or young person
CHAQ: Childhood Health Assessment Questionnaire
CHU 9D: Child Health Utility 9 Dimensions
HRQoL: Health related quality of life
VAS: Visual analogue scale
SoC: Standards of care
OMERACT: Outcome measures in rheumatology
