# Supplementary material for: Validation of novel patient-centred juvenile idiopathic arthritis-specific patient-reported outcome and experience measures (PROMs/PREMs)
Source: Pediatr Rheumatol Online J. 2020 Nov 19;18:91. doi: 10.1186/s12969-020-00481-2 (PMC7678156; doi:10.1186/s12969-020-00481-2)
Supplement: Supplementary file 1 — Additional file 1: Supplementary Table S1. Patient Reported Outcome and Experience Measures for Children Aged < 11 Years. Supplementary Table S2. Patient Reported Outcome and Experience Measures for Young People Aged ≥ 11 years. [file 12969_2020_481_MOESM1_ESM.docx]

**Supplementary Table S1: Patient Reported Outcome and Experience Measures for Children Aged < 11 Years**

| **Patient Outcome** | **Never** | | **Sometimes** | | | **Often** | | **Most of**  **the time** | |
| --- | --- | --- | --- | --- | --- | --- | --- | --- | --- |
| ***Question 1: Physical well being***  Over the past month, how often has each of the following interfered with your child’s daily activities?  Fatigue (having very little energy)  Pain  Poor sleep  Medication (side effects) | 🞏  🞏  🞏  🞏 | | 🞏  🞏  🞏  🞏 | | | 🞏  🞏  🞏  🞏 | | 🞏  🞏  🞏  🞏 | |
| ***Question 2: Social Wellbeing***  Over the last month, how often has your child’s condition interfered with the things your child like(s) to do (e.g. playing sport, going to the park, playing out, socialising with friends). | 🞏 | | 🞏 | | | 🞏 | | 🞏 | |
| ***Question 3: Emotional Wellbeing***  Over the last month, how often has your child felt sad/worried or frustrated? | 🞏 | | 🞏 | | | 🞏 | | 🞏 | |
|  | | | | | | | | | |
| **Patient Experience** | **Not**  **at all** | | **A bit** | | | **Mostly** | | **Fully** | |
| ***Question 1****:*  During today’s hospital visit, were your questions and concerns listened to and answered in a way that you could understand? | 🞏 | | 🞏 | | | 🞏 | | 🞏 | |
| ***Question 2****:*  How well do you understand your treatment plan? | 🞏 | | 🞏 | | | 🞏 | | 🞏 | |
| ***Question 3****:*  How well supported do you feel in between hospital visits? | 🞏 | | 🞏 | | | 🞏 | | 🞏 | |
| ***Question 4***:  Was the environment in which you waited today suitable for you and your family (those attending the appointment) | 🞏 | | 🞏 | | | 🞏 | | 🞏 | |
| **Patient Experience** | **No delay** | **< 15 mins** | | **15-30 mins** | **30-60 mins** | | **1-2 hours** | | **>2 hours** |
| ***Question 5****:*  From your appointment time today, if you experienced a delay, please tell us how long you were delayed? | 🞏 | 🞏 | | 🞏 | 🞏 | | 🞏 | | 🞏 |
| **Question 6:**  Do you feel your delay was unacceptable? Yes 🞏 No 🞏 Not applicable 🞏 | | | | | | | | | |

**Supplementary Table S2: Patient Reported Outcome and Experience Measures for Young People Aged ≥ 11 years**

| **Patient Outcome** | **Never** | | **Sometimes** | | | **Often** | | **Most of**  **the time** | |
| --- | --- | --- | --- | --- | --- | --- | --- | --- | --- |
| ***Question 1: Physical well being***  Over the past month, how often has each of the following interfered with your daily activities?  Fatigue (having very little energy)  Pain  Poor sleep  Medication (side effects) | 🞏  🞏  🞏  🞏 | | 🞏  🞏  🞏  🞏 | | | 🞏  🞏  🞏  🞏 | | 🞏  🞏  🞏  🞏 | |
| ***Question 2: Social Wellbeing***  Over the last month, how often has your condition interfered with the things you like to do (e.g. playing sport, going to the park, playing out, socialising with friends). | 🞏 | | 🞏 | | | 🞏 | | 🞏 | |
| ***Question 3: Emotional Wellbeing***  Over the last month, how often have you child felt sad/worried or frustrated? | 🞏 | | 🞏 | | | 🞏 | | 🞏 | |
|  |  | |  | | |  | |  | |
| **Patient Experience** | **Not**  **at all** | | **A bit** | | | **Mostly** | | **Fully** | |
| ***Question 1****:*  During today’s hospital visit, were your questions and concerns listened to and answered in a way that you could understand? | 🞏 | | 🞏 | | | 🞏 | | 🞏 | |
| ***Question 2****:*  How well do you understand your treatment plan? | 🞏 | | 🞏 | | | 🞏 | | 🞏 | |
| ***Question 3****:*  How well supported do you feel in between hospital visits? | 🞏 | | 🞏 | | | 🞏 | | 🞏 | |
| ***Question 4***:  Was the environment in which you waited today suitable for you and your family (those attending the appointment) | 🞏 | | 🞏 | | | 🞏 | | 🞏 | |
| **Patient Experience** | **No delay** | **< 15 mins** | | **15-30 mins** | **30-60 mins** | | **1-2 hours** | | **>2 hours** |
| ***Question 5****:*  From your appointment time today, if you experienced a delay, please tell us how long you were delayed? | 🞏 | 🞏 | | 🞏 | 🞏 | | 🞏 | | 🞏 |
| **Question 6:**  Do you feel your delay was unacceptable? Yes 🞏 No 🞏 Not applicable 🞏 | | | | | | | | | |
